# Where are the African voices and perspectives of women in sport coaching?

**DOI:** 10.3389/fspor.2022.909965

**Published:** 2022-09-15

**Authors:** Nana Akua Achiaa Adom-Aboagye

**Affiliations:** Centre for Sport Leadership, Maties Sport, Stellenbosch University, Stellenbosch, South Africa

**Keywords:** Africa, female coaches, South Africa, sport coaching, women

## Introduction

With sport recognized as a global phenomenon, it would be reasonable to assume that current studies and research would reflect the diversity and complexity within this field and its various branches of representation. However, what research often depicts and projects is the continued marginalization and under-representation of Global South scholarship as well as limited representation of sport's heterogeneity, particularly surrounding women and sport (Adom-Aboagye and Burnett, 2019). This is reflective of sociological trends and theories that broadly portray Global North perspectives, whilst positioning support for post-colonial knowledge production. This could in part be attributed to a (unintended) superficial understanding of indigenous knowledge and experiences (Connell, 2007) by Global North scholars.

Current studies on black African women and their lived experiences are limited. My own research has shown that the sociology of women in sport mostly produces and disseminates knowledge related to Global North experiences, leaving out black (African) women living in the Global South (Adom-Aboagye and Burnett, 2019). Their voices and experiences are scarce, pointing to a clear need within sport and gender studies research to attend to the intersections of race, gender, nationality and region.

My own positionality is that of an early career black African scholar. At the start of my academic career nearly a decade ago, I noted that although scholarly output on various women and sport debates and issues provided good foundational arguments and recommendations, they usually did not reflect the true lived experiences of black African woman in sport (Burton and Leberman, 2017; Evans and Pfister, 2021). These are indeed experiences that world-renowned scholars have recently admitted need further exploration and inclusion (Burton and Leberman, 2017; Evans and Pfister, 2021). In response to this call, I use this research essay to undertake an intersectional analysis of how sociological knowledge is being produced on women in sport coaching, a topic of increasing interest to feminist sociologists working in the domain of sport (Kane, 2016; LaVoi et al., 2019).

With respect to women in sport coaching in Africa, there is currently no central database that provides a statistical account of women and their representation across various levels and sports in Africa. One would have to undertake the laborious work of a sport-by-sport analysis in order to investigate the gender representation within its coaching structures, with limited research funding available to do so in Africa. Such work is primarily possible at elite levels of sport coaching and not necessarily at lower levels of coaching. As an example of women's under-representation in African coaching: at the Women's Africa Cup of Nations football tournament staged in Morocco in July 2022, only three of the 12 teams were coached by women (CAF Online, 2022a,b,c; Soccerway, 2022; SuperSport, 2022). Two further countries replaced their women coaches with men soon after qualifying for the tournament. By contrast, the Women's European football tournament, which was held concurrently, has six female coaches, out of 16 teams being represented (UEFA, 2022). Although this is a slight increase in comparison to the African teams, we still need to understand the specific factors that hamper the advance of female coaches in Africa, without relying too much upon Global North research to tell a master narrative about “the” experience of women in sport coaching.

Within the realms of feminist thought and sociological debates, intersectionality was born from the need to holistically represent the differences amongst women, especially amongst women of colour and the continued marginalization and inequality that the feminist movement unintentionally masked (Hayes, 2017). Institutionally, intersectionality is often reflected in the drive to promote diversity, equity and inclusion (DEI) across society.

Sport is one such sub-sector of society where the calls for diverse and equitable representation have been strong. The formalization of the women and sport movement in 1994 is one such example (Matthews, 2014). The women and sport movement and historical moments galvanized sport as a social development agenda (Kidd, 2008; Darnell et al., 2019), also helping position the fledgling field of sociology of sport as a scholarly field that could provide inclusive and equitable knowledge outputs (Schulenkorf et al., 2016; Darnell et al., 2018). Yet such studies often lack critical self-reflection and an acknowledgement that the eventual output lacks indigenous knowledge reflections, considerations and contributions from scholars and advocates within regions beyond the Global North (Toffoletti et al., 2018).

Thus, the purpose of this research essay is to highlight the unexplored dynamics of women in sport coaching in scholarly research. I argue that Global South and African knowledge production can provide context specific contributions to women in sport coaching research, that could be of benefit to Global North scholars. As Global South scholars, if given the chance, we can offer original—and indeed necessary—insights into the dynamics of inequality and exclusion that continue to characterize global sport issues.

## Methodological approach

The method used in this review of the existing literature is motivated by Evans and Pfister (2021) systematic narrative review of women in sports leadership. Evans and Pfister's review recognized the over-representation of Global North perspectives in women in sports leadership research. The question that guided my review of the women in sport coaching literature was: *What evidence exists regarding the extent to which African experiences and perspectives are represented in studies examining women in sport coaching?*

Data collection involved the utilization of EBSCOhost Web, made available through my institutional library. The search resulted in 60,400 hits, using a combination of the following search terms: women, female, coaching, coach, gender inequality, gender equity, gender equality and sport. This search process was refined, using Boolean search strings, limiting searches to publications (articles, book reviews, book chapters, news articles and opinion pieces) after 1994—the year the *Brighton Declaration* was adopted. This adaptation produced 1634 hits, and was later narrowed down to 592 articles, when I factored in English only publications. The removal of duplicates left me with 367 publications. I scanned the publications, focusing on female coaches (as subjects under investigation—wholly or in part) and their experiences. This left me with 124 publications, of which I could only access 101 for download. Secondary searches were conducted on WorldCat (which garnered no new publications) and Google Scholar (which garnered 24 new publications). This left me with 125 publications to review. Of the 125 publications, 65 were from the United States; 29 were from Europe; 18 were from Canada; nine were from the Asia Pacific and four were from Africa (South Africa).

## Representation of women in sport coaching

A review of the literature highlighted four main recurring themes concerning women in sport coaching: stereotypes and misconceptions; lack of knowledge; cultural expectations and family challenges and opportunity and structural barriers. Misconceptions on a woman's ability to coach stemmed from gender stereotypes. Stereotypes that posit that because female sport is deemed less professional than male sport, and there being fewer opportunities for women to reach elite levels of competition than men—women are therefore not as knowledgeable and/or competent as their male peers in coaching (Kilty, 2006). Lack of knowledge within coaching for women, has been linked to the limited amount of time women are perceived to spend in sport in relation to men, due to societal expectations. Expectations that have been superimposed by cultural expectations and family challenges. Most of the publications referring to cultural expectations, pinned their arguments on gendered cultural norms and their traditional gender role expectations (Wicker et al., 2019). Often, these are expectations that make women feel guilty for pursuing coaching excellence if they have families and paradoxically has them questioning themselves and their womanhood if they do not have families (Norman, 2014), in lieu of coaching. These issues all converge upon structural barriers and the opportunities (or lack thereof) that they generate.

It is well known that men still hold the seats of power, access and resources in sport (Fisher, 2019). Thus, it can be argued that patriarchal hegemony still permeates sport structures. And with stagnated gender reform at this level, it is not surprising that there continues to be a lack of opportunity and support for women in coaching (Norman, 2008). However, research suggests that such dynamics are even more complicated in the African region, where women face additional patriarchal, hegemonic and misogynistic challenges to autonomy, such as femicide, child marriages, female genital mutilation and inheritance rights issues (Demirgüç-Kunt et al., 2013; Petroni et al., 2017; Obiora et al., 2020; Boonzaier, 2022).

The highlighted themes from Global North scholars are generic, yet relatable to women in coaching from various regions of the globe. However, with the global drive towards DEI and greater representation, only a few of the publications reviewed touched on race and/or class as an additional layer to the challenges faced by women in sport coaching (Thomas, 2006; Walker and Bopp, 2011), with no references to the African context. With other publications, there was not enough data obtained to represent the influence of intersectionality on women in sport coaching (Norman and Rankin-Wright, 2018; Fisher, 2019), especially a developing view.

As previously stated, there were only four publications from (South) Africa, and they acknowledged the generic themes that I have presented (in varying forms) (Surujlal and Vyas-Doorgapersad, 2015; Kubayi et al., 2017a,b, 2020). These four publications bridged over a period of 5 years (2015–2020), representing at least some contribution from a non-Global North context to this important field. The authors acknowledged where their research converged with international scholars and contributed information that global publications did not highlight (Kubayi et al., 2020).

What these studies discovered was that in South Africa, most female coaches were volunteers, often receiving minimal stipends and if they were paid, it was generally not enough to subsist upon alone (Kubayi et al., 2017b, 2020). This can be attributed to most (female) coaches in South Africa not having formal coaching qualifications (not a formalized requirement in the country currently), which was identified as impacting upon their level of remuneration and career advancement opportunities, irrespective of their years of experience (Kubayi et al., 2017a, 2020). All of which culminates in experiences of job insecurity for female sport coaches in South Africa (Surujlal and Vyas-Doorgapersad, 2015). By comparison, more training, development and professional opportunities appear to exist for female coaches in the Global North, although still under conditions of inequality.

A further point here that is important to emphasize: research participants in the above four South African publications were mostly black African, whereas previous publications reflected mostly white participants. This is significant, because we cannot always assume that research from the Global South is always inherently inclusive. A country like South Africa for instance has a history of white supremacy (apartheid) and this has had in influence on knowledge making institutions within and outside the country.

In addition, there have been numerous studies from the Global North on the experiences of black women coaches in sport (Borland and Bruening, 2010; Carter-Francique and Olushola, 2016; Rankin-Wright et al., 2019). Although relevant to the body of knowledge being produced, such studies are not representative of the experiences of black African women, who often face differing sociological challenges as to their Global North counterparts (Demirgüç-Kunt et al., 2013).

## Discussion and conclusion

The literature reviewed shows a gap in scholarly output with respect to the jointly racial and regional representation of women in sport coaching. Bar the South African examples, research thus far has not gone beyond the commonly recognized barriers of patriarchy, misogyny, pay inequality etc. experienced by female coaches (Norman, 2008; Fisher, 2019; Kraft et al., 2020). This highlights the need to explore and investigate the nuances relating to power imbalances and inequalities experienced amongst female coaches of different classes, races, nationalities, sexual orientations and disability. Nuances in the era of DEI, that seem to forget that women are not a homogenous group within sport, especially women of colour. For example, Norman (2014) touches upon race and culture, but does not delve into unpacking its intricate complexities, especially for women of colour in a Global North country. If she had, this would have provided an opportunity for an exploration or comparative analysis of the differences and similarities that race and culture creates amongst female coaches in developed and developing nations.

As a scholar of colour located in the Global South, I am evidence that there are scholars who can provide feminist, decolonial contributions to global knowledge production within sport. It is not that we don't exist; rather, it is perhaps that our views and writing styles do not subscribe to Westernised output and expectations. Region and/or nationality of scholarly origin and the high costs of research and publications (Roberts and Connell, 2016), also contribute to impeding Global South and specifically African knowledge production. These impediments likely also impact upon the production of knowledge around women in sport coaching.

Future research studies would also do well to utilize appropriate feminisms from the various regions of study that speak to the women under investigation, particularly for groups of identified women whose voices are absent in women in sport coaching literature. For example, the studies reviewed and touched on above, would not be entirely appropriate as terms of reference for the under-developed African context, as they are devoid of the lived experiences of black African women within their unique patriarchal systems. These systems have been influenced by colonial trends (Morountodun, 2019), poverty, oppressive traditions, cultures, structures and practices (Chiweshe, 2018)—which now find themselves intersecting with recent decolonial demands. Moreover, and taking heed of the insight from postcolonial scholars like Mohanty (1988) and Spivak (1988), who posit that Global North representations tend to homogenize women living in the Global South, as scholars, we should look to discover the diversity of experiences for women coaches who live and work on the African continent.

It is one thing to *speak of* intersectionality and its importance to scholarly output. However, the greater challenge is to ensure that scholarly output *reflect*s the heterogenic make-up that is global women in sport coaching. This can be attributed to intersectional erasure—which is perhaps unintentional, but has effects nevertheless. If the experiences of black African women coaches are not researched and reported upon going forward, (akin to studies in the Global North), views and outputs for gender and sport studies will continue to be skewed towards a specific point of view. This is not just in opposition to continued calls for intersectional representation: it undermines the knowledge base that we rely on to challenge the entrenched under-representation of women in coaching and other areas of sports administration and leadership.

## Author contributions

The author confirms being the sole contributor of this work and has approved it for publication.

## Conflict of interest

The author declares that the research was conducted in the absence of any commercial or financial relationships that could be construed as a potential conflict of interest.

## Publisher's note

All claims expressed in this article are solely those of the authors and do not necessarily represent those of their affiliated organizations, or those of the publisher, the editors and the reviewers. Any product that may be evaluated in this article, or claim that may be made by its manufacturer, is not guaranteed or endorsed by the publisher.
